# Supplementary material for: BAP31 Knockout in Macrophages Affects CD4+T Cell Activation through Upregulation of MHC Class II Molecule
Source: Int J Mol Sci. 2023 Aug 30;24(17):13476. doi: 10.3390/ijms241713476 (PMC10487781; doi:10.3390/ijms241713476)
Supplement: Supplementary file 1 [file ijms-24-13476-s001.zip › ijms-2525363-supplementary.pdf]

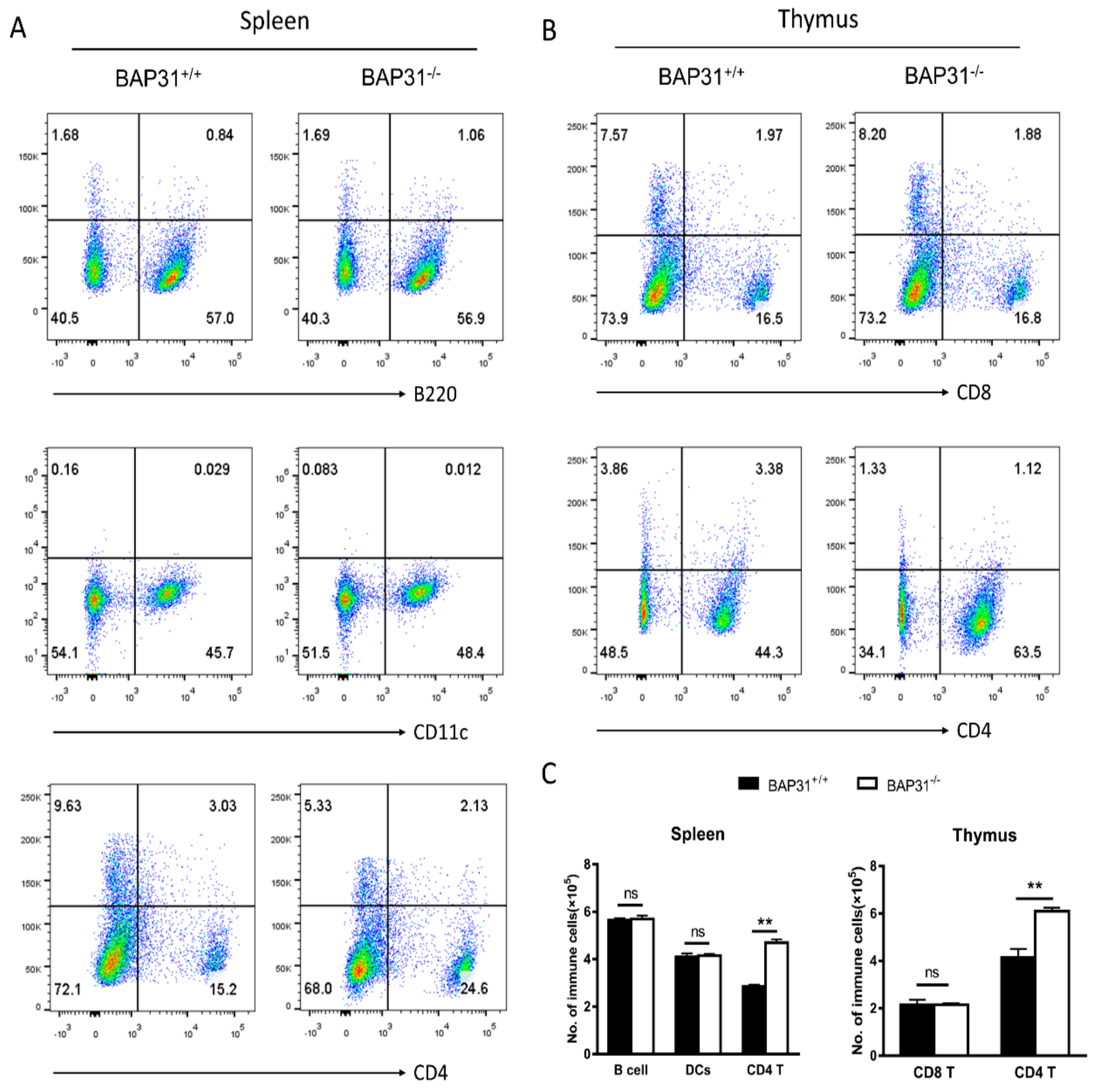

Supplementary Figure S1. (A) Flow cytometry detect the immune cells of splenocyte from BAP31$^{flox/flox}$ mice (BAP31$^{+/+}$) and BAP31$^{flox/flox}$Lyz2-cre mice (BAP31$^{-/-}$) (n=3). (B) Flow cytometry detect the immune cells of thymocyte from BAP31$^{flox/flox}$ mice (BAP31$^{+/+}$) and BAP31$^{flox/flox}$Lyz2-cre mice (BAP31$^{-/-}$) (n=3). (C) Statistical bar charts showing the cell numbers of immune cells of splenocyte and thymocyte from BAP31$^{flox/flox}$ mice (BAP31$^{+/+}$) and BAP31$^{flox/flox}$Lyz2-cre mice (BAP31$^{-/-}$) (n=3). **P<0.01. ns, no significant difference.

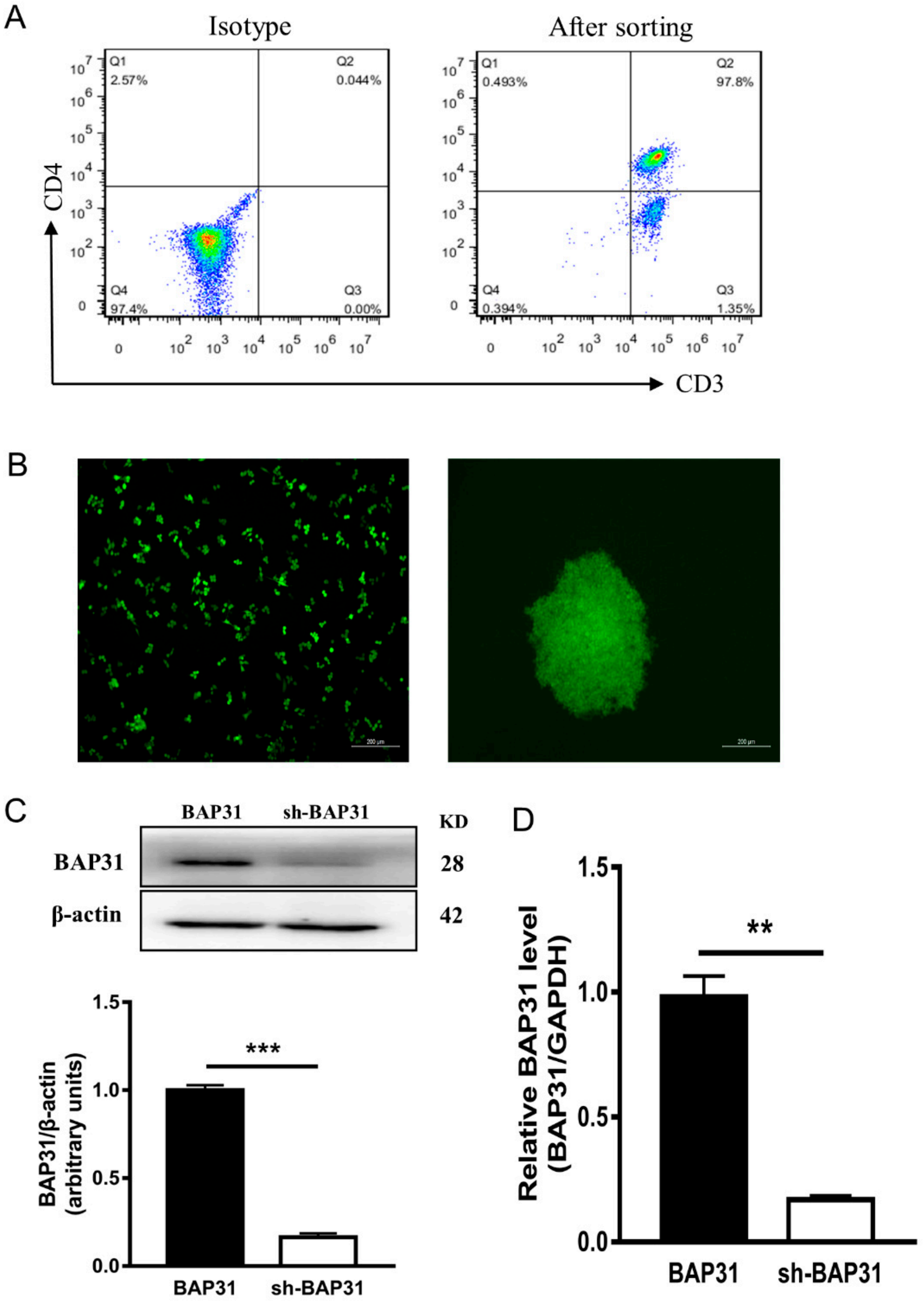

Supplementary Figure S2. (A) Flow cytometry detect the purity of naïve CD4⁺T cell obtained from mouse spleen cells by magnetic bead sorting. (B) Fluorescence microscope observe the knockout BAP31 stable transfected RAW264.7 cells and obtain the monoclone cell line. (C) Western blotting verify the knockout efficiency of BAP31 at protein level(n=3). Relative protein expression is expressed as the ratio of the BAP31 to β-actin. (D) RT-qPCR analysis the knockout efficiency of BAP31 at mRNA level(n=3). Relative BAP31 expression was normalized by GAPDH expression. ** P<0.01. ***P<0.001.

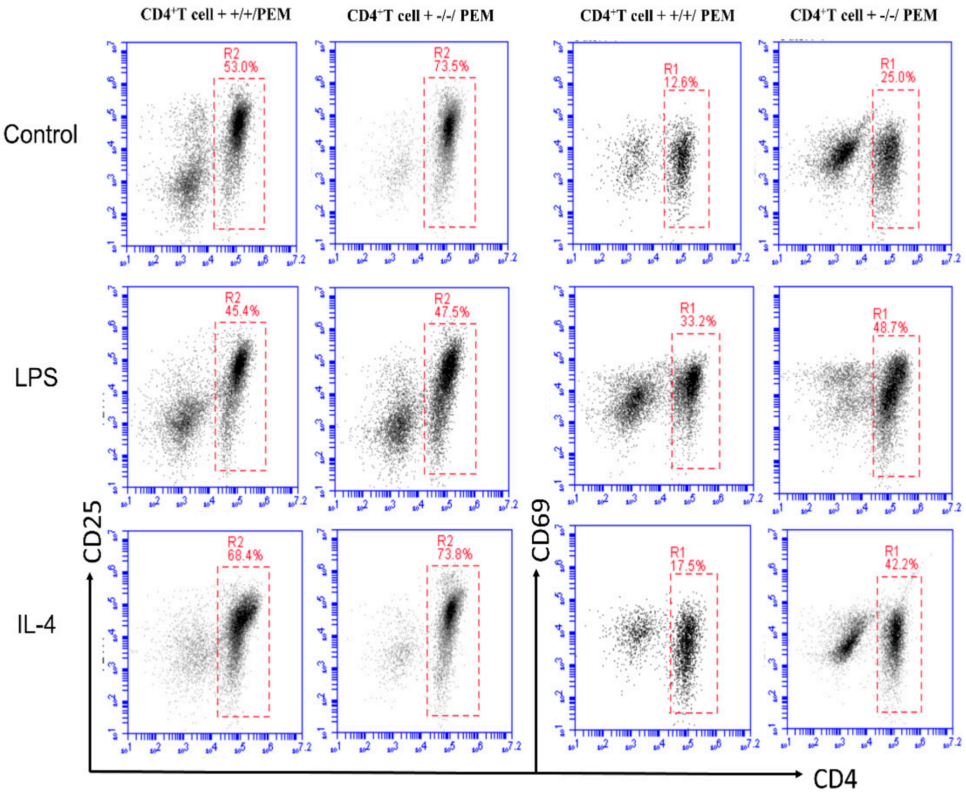

Supplementary Figure S3. Flow cytometry detect T cell activation markers (CD25, CD69) of naïve CD4$^+$T cell co-culture with peritoneal macrophages 72h divided control, LPS and IL-4 group.

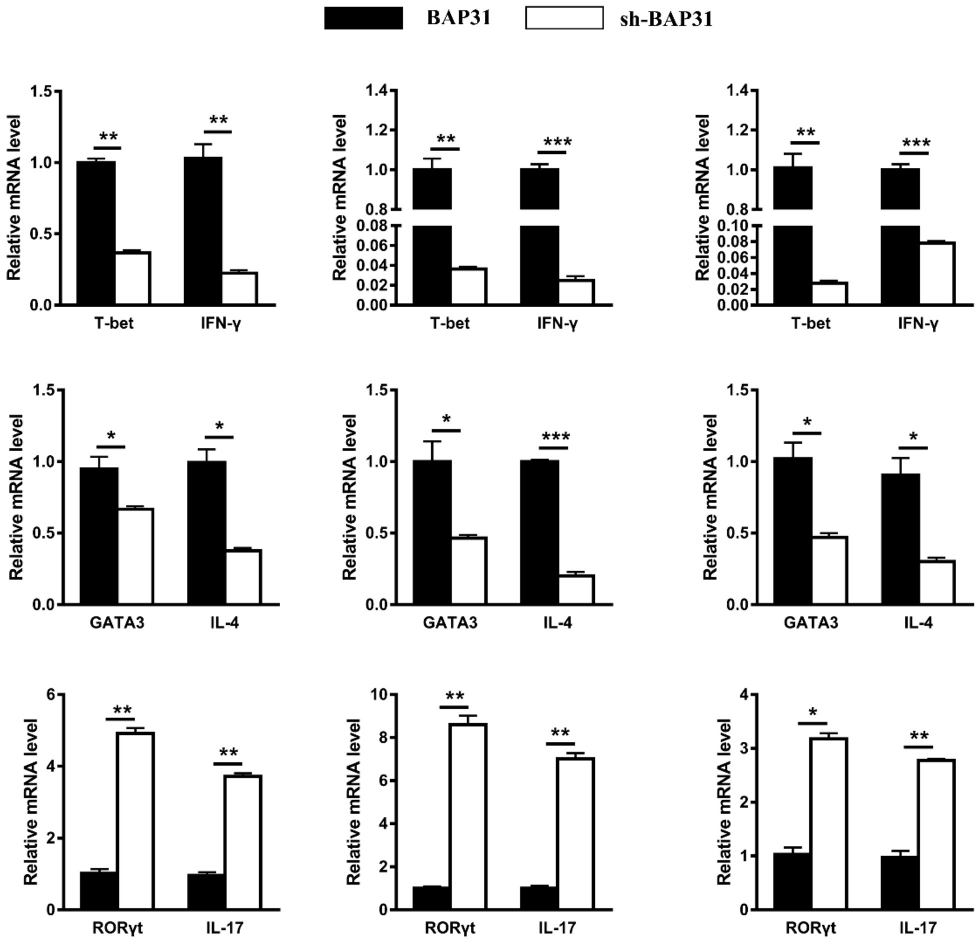

Supplementary Figure S4. Real-time PCR analysis differentiation of CD4^{+}T cell subset Th1(IFN-γ), Th2(IL-4), Th17(IL-17A). EL4 cells co-culture with RAW264.7 cells 72h divided control, LPS and IL-4 group(n=3). *P<0.05.P<0.01. ***P<0.001.**
